# The impact of saliva collection and processing methods on CRP, IgE, and Myoglobin immunoassays

**DOI:** 10.1186/2001-1326-1-19

**Published:** 2012-09-05

**Authors:** Roslinda Mohamed, Jennifer-Leigh Campbell, Justin Cooper-White, Goce Dimeski, Chamindie Punyadeera

**Affiliations:** 1 The Australian Institute for Bioengineering and Nanotechnology, St-Lucia, Australia; 2 The School of Chemical Engineering and the University of Queensland, Queensland, Australia; 3 Chemical Pathology, Princess Alexandra Hospital, Pathology Queensland, Woolloongabba, Australia; 4 School of Medicine, Southside Clinical School, The University of Queensland, Herston, Australia; 5 Saliva Translational Research Group, Tissue Engineering and Microfluidic Laboratory, the Australian Institute for Bioengineering and Nanotechnology, The University of Queensland, Old Cooper Road, St. Lucia, Queensland, 4072, Australia

**Keywords:** Human saliva, Homogeneous bead-based assay, Salivary stimulations, Salivary pre-processing techniques, Non-invasive

## Abstract

**Background:**

Owing to its ease of collection, saliva is potentially the sample of choice in diagnosis. Salivary biomolecules have provided a porthole in surveying a person’s health and well-being. Our study aims were (1) to demonstrate the effects of pre-analytical steps, collection and pre-processing techniques on salivary protein detection and (2) to establish an indication of salivary reference intervals for 3 biomolecules of clinical interest.

**Methods:**

Saliva samples were collected from participants (n = 25, ages 20–35 years) using the following methods: no stimulation (resting/unstimulated), mechanical, and acid stimulation. The saliva was prepared for analysis by: unprocessed, post standard centrifugation in a container without any additives, and centrifugation using Centrifugal Filter Unit (Amicon® Ultra-0.5). AlphaLisa® assays were used to measure the levels of C-Reactive Protein (CRP), Immunoglobin (IgE) and myoglobin in saliva samples.

**Results:**

Saliva flow rates were lowest with the resting/drooling collection method. The lowest total protein concentration was with acid stimulation. Unstimulated and mechanically stimulated collections produced no effect on the CRP and IgE levels while myoglobin levels were highest with the unstimulated collection. Acid stimulation had a negative impact on the measured concentrations of IgE and myoglobin (except for CRP levels).

**Conclusion:**

Mechanical stimulation was the most viable option for collecting saliva without affecting the levels of CRP and myoglobin. The processing methods had an adverse effect on the concentration of total protein as well as on CRP and IgE concentrations.

## Background

Human saliva is a unique biological fluid with numerous functions within the oral cavity, predominantly facilitating the maintenance of oral health and creating an appropriate ecological balance in the mouth [1]. Human saliva mirrors the body’s health and wellbeing and approximately 20-30% of proteins [2,3] found in blood are also present in saliva, highlighting the diagnostic potential of this biological matrix. The advantages of using saliva as a diagnostic body fluid compared to blood are (a) sampling is non-invasive, rapid and allows multiple sample collections; (b) the collection process is relatively simple, safer and painless and ideal for population based screening programs; (c) sampling can be carried out by patients or carers to facilitate self-management of disease monitoring at home or care/clinical setting as well as in challenging climates; (d) the method of collection does not require a skilled workforce thereby reducing costs associated with sampling; and (e) there is minimal threat to the collector of contracting infectious agents, such as Hepatitis and or HIV, through handling saliva [2,4-11].

Saliva is produced by three major salivary glands (parotid, submandibular and sublingual) and numerous minor salivary glands. Saliva contains a myriad of salivary proteins which could serve as biological markers for diagnosing and tracking the progression of various health conditions, as well as monitoring the effectiveness of medication [8,12,13]. To date, most of the saliva collection devices that are commercially available allow a person to collect resting/unstimulated saliva and/or stimulated saliva either via mechanical stimulation or acid stimulation [5,6]. When a person is in a resting state, saliva production is largely produced by the submandibular gland, while only 20% and 8% are produced by parotid and sublingual glands, respectively [14,15]. In contrast, when saliva production is stimulated either via chewing gum or plastic (e.g. parafilm), or through acid stimulation, most of the saliva produced is primarily derived from the parotid gland [14,16]. Most importantly, the composition of both stimulated and unstimulated saliva may be altered by genetic predisposition factors and physiological, pathological and environmental factors [14,15,17,18], all these factors may hinder the correct derivation of results for best care outcomes.

Most of the proteins that are present in saliva are either synthesized *in situ* in the salivary glands and/or transported from blood capillaries into saliva by diffusion, active transport and/or ultra-filtration [1,5]. The proteins in saliva may also undergo modifications (glycosylation, deglycosylation, phosphorylation) due to underlying pathological conditions and/or as a result of exposure to drugs and other compounds or solutions. Our understanding of the biomolecules present in saliva during a normal healthy physiological state, as opposed to a pathological condition, requires further investigation in order for saliva to become a sample of choice for diagnostic and treatment purposes.

Current obstacles to the translation of saliva from a laboratory to the bench side of a patient are: (a) the low concentration of analytes (100 to 1000 fold lower) in saliva compared with blood, which requires more sensitive detection/measurement technologies; (b) concentration levels and composition may be influenced by diurnal/circardian cycles; (c) the type of saliva collection and processing methods [5,8].

The aims of this study were a) to investigate the impact of collection methods (resting, mechanical and acid stimulation), and processing techniques on three analytes with varying sizes (C-Reactive Protein (CRP) 115 kDa [19], Immunoglobulin E (IgE) 160 kDa [20], and myoglobin 16.7 kDa [21] measured by immunoassay techniques, b) to provide an estimate or guide on the potential salivary reference intervals for the suggested collection and processing methods. The findings from our work can be extrapolated onto other biomolecules of interest in saliva with similar molecular sizes.

## Methods

### Participants

The study was approved by The University of Queensland Medical Ethical Institutional Board and all participants signed an informed consent. We recruited 25 healthy participants, 12 females (18–34 years) and 13 males (18–29 years). The exclusion criteria obtained via a questionnaire included no intake of any type of drugs/medicines; existence of any co-morbid and/or oral disease (e.g. periodontal disease and gingivitis), autoimmune, infectious, musculoskeletal, or malignant disease, and recent operation or trauma. In addition, participants had to be free of fever and/or cold, non-smokers and had good oral hygiene. Participants were asked to refrain from eating and drinking two hours prior to saliva collection in order to obtain a relatively constant baseline.

### Saliva stimulation methods

Three saliva collection techniques were assessed drool (resting), mechanical and acid stimulations. All the saliva samples were collected between 9 am - 12 pm to minimise diurnal variations associated with saliva sampling. In addition, both females and male participants were <35 years of age to minimise age related differences in the salivary biomolecular composition [22,23]. The participants rinsed their mouth with water prior to collection, and waited 10 minutes before commencing with the collection. Participants were allowed 5–10 minutes between different collections to avoid cross contamination of saliva between collections. The collection order was resting, followed by mechanical stimulation and lastly, acid stimulation.

Resting drooling (minimal oral movements) was used to collect whole mouth saliva from the oral cavity [24,25]. Participants were asked to sit comfortably in an upright position and tilt their heads down slightly to pool saliva in the mouth. The first expectoration was discarded to eliminate food debris and unwanted substance contaminating the sample that may cause analytical inaccuracy. The subsequent sample was then expectorated into a pre-labelled sterile container and ~ 2 mL saliva was collected. The samples were then immediately placed on ice to minimise degradation of salivary proteins until further processing.

In order to obtain mechanically stimulated saliva, the participants were asked to chew onto a piece of tubing (200 mm cut pieces of 1.6 mm bore, 1.4 mm wall platinum cured silicone tubing) for 1 minute and then expectorate the first component while keeping the tubing in the mouth. The participants expectorated the saliva into a pre-labelled sterile container once every 30 seconds while chewing onto the tubing until an adequate volume (~2 mL of saliva) was collected. The samples were then immediately placed on ice to minimise bacterial degradation of salivary proteins until further processing. In order to collect acid stimulated saliva, food-grade citric acid (0.25%, supermarket grade) was prepared and participants were asked to swirl 5 mL of the citric acid solution in the mouth for 15 seconds and then expectorate into a waste container. This procedure was repeated. The participants were then asked to pool saliva in their mouth and expectorate every 30 seconds into a pre-labelled sterile container until ~2 mL of saliva was collected and processed on ice similar to the above procedure. Salivary flow rate was calculated by dividing the saliva volume (mL, measured using a pipette) by the time (mins) it took to produce the volume of saliva measured.

### Pre-processing techniques

All salivary samples were subjected to freeze–thaw cycle to break down mucopolysaccharides to reduce viscosity and to minimise pipetting errors [6]. The unprocessed samples were aliquoted directly into 1.5 mL Eppendorf tubes and stored at -80°C. All thawed saliva samples were centrifuged (10,000 × g for 10 minutes at 4°C) to remove cellular debris and to minimise the turbidity of saliva, which can negatively impact on the accuracy of analysis [26]. The supernatant was transferred into a fresh Eppendorf tube and appropriately labelled. The saliva samples were centrifuged using Centrifugal Filter Units (Amicon Ultra 30 K centrifugal filter devices, Millipore, USA) as a means of concentrating low MW analytes present in saliva. The samples were processed using Centrifugal Filter Units as per manufacturer’s instructions. Following pre-processing, all samples were frozen at −80°C until analysis.

### Quantification of total salivary proteins

Bicinchoninic acid (BCA) protein assay (Thermo Scientific, IL, USA) was used to determine the total protein concentration in human saliva. The assay was performed in a 96-well micro plate according to the manufacturer’s protocol, using 10 μL of each unknown sample and standard. The plates were read at 562 nm using SpectroMax plate reader (Molecular Devices, CA, USA).

### Quantification of salivary CRP, IgE and myoglobin levels

AlphaLISA® kits (Perkin Elmer®, MA, USA) were used to determine CRP (#AL233C), IgE (#AL292C) and myoglobin (#AL285C) concentrations in the saliva. These protocols have previously been optimized by the manufacturer for the optimal concentration of acceptor beads and biotinylated antibody concentrations. The total sample volume used for each assay was 10 μL. Twelve standard assay points were used to generate a standard curve. The samples were analysed in triplicate in 384 well ProxiPlates™ (Perkin Elmer®, MA, USA). The only exception to manufacturer recommendations was the decrease in the total reaction volumes from 50 μL to 10 μL. In summary, the assay consisted of undiluted saliva sample/analyte (1 μL), biotinylated antibody (10 mM) and acceptor bead (40 μg/mL) mix and streptavidin donor beads (50 μg/mL). For all assays, the end concentration of acceptor beads was 10 μg/mL whilst the end concentration of biotinylated antibody was 1nM. The total incubation time was 1.5 h at room temperature in the dark and the plates were read using an EnSpire™ plate reader (Perkin Elmer®, MA, USA). Intra- and inter-assay coefficients of variation (CVs) for all the above-mentioned saliva assays were below 5%.

### Statistical analysis

All statistical analyses were performed using GraphPad Prism software version 5.04 (GraphPad Software Inc., USA). Standard curves were generated using a 4-parameter logistic equation (sigmoidal dose–response curve with variable slope) and a 1/Y2 data weighting [27].

The measurements obtained for the study were evaluated for normal distribution using the D'Agostino and Pearson omnibus normality test and found the data to be non-Gaussian distributed. The Wilcoxon matched-pairs signed rank test was used to compare data generated using the three collection methods. Statistical significance between the collection methods were assessed as p <0.05 and was calculated using the same program.

## Results

### Salivary flow rates in healthy volunteers according to stimulation method

The salivary flow rates in the study participants for resting, acid and mechanically stimulated saliva were 0.52 ± 0.22, 0.79 ± 0.34 and 1.41 ± 0.61 mL/min respectively. Unstimulated/resting saliva collection gave the lowest (p <0.05) mean salivary flow rate when compared with the stimulated methods. Saliva samples stimulated by mechanical stimulation produced significantly (p <0.05) higher salivary flow rate when compared with unstimulated resting drool.

### Salivary total protein concentrations in healthy volunteers

**Figure 1:**
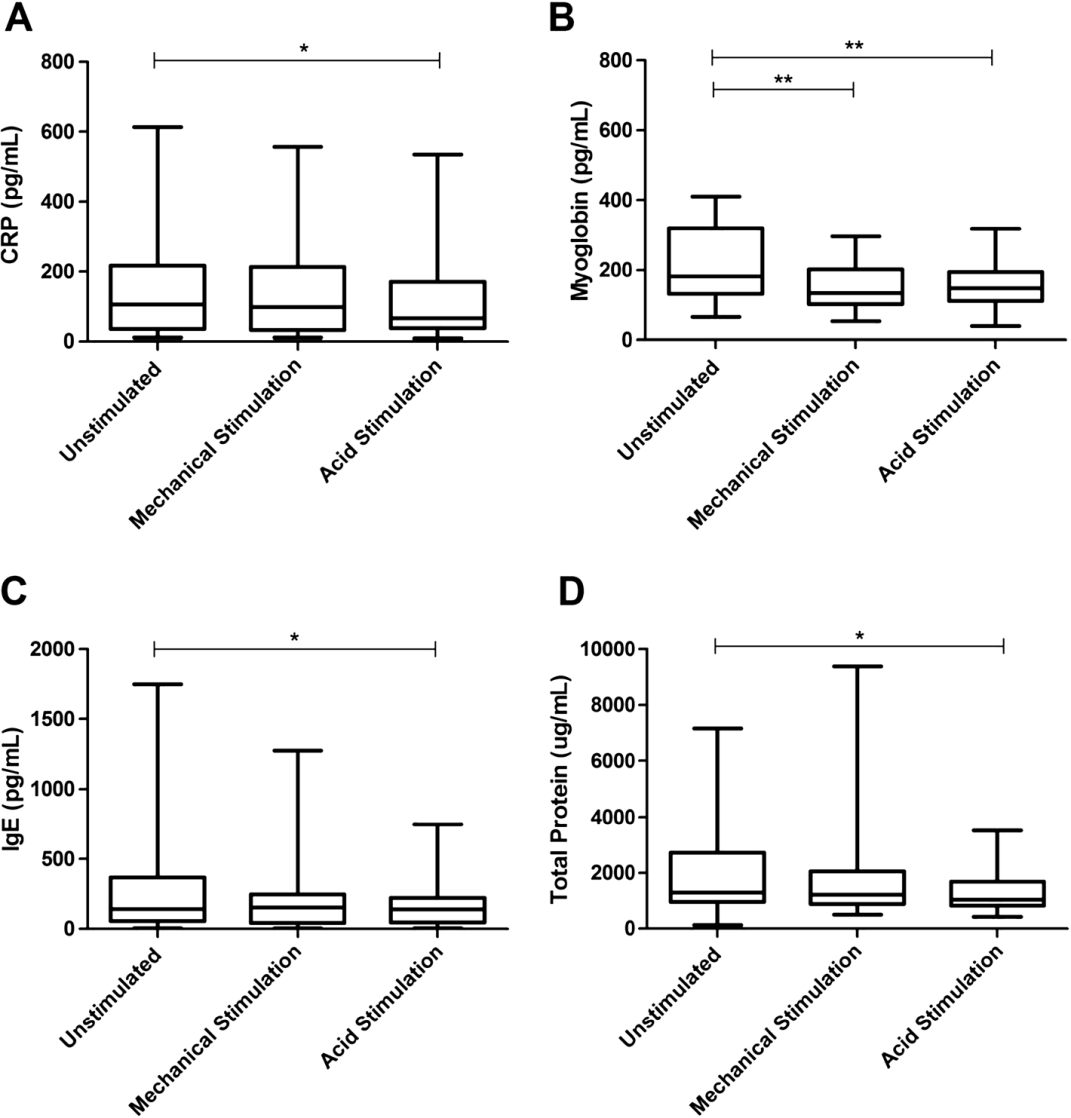
**Concentration of (A) CRP, (B) Myoglobin, (C) IgE and (D) Total Protein in the resting drool, mechanically stimulated drool and acid stimulated drool samples. **Minimum and maximum data values are indicated by bottom- and top-most points of box plot. Lower quartile, median and upper quartile values are indicated by first, second and third horizontal lines of the box in the box plot. Significance is shown as *p <0.05,**p <0.01, ***p <0.001.

The total protein concentration range for the participants is shown in Tables 1 and 2. The saliva pre-processing technique (*i.e.* centrifugation) significantly reduced the total protein levels in the saliva samples. The total protein concentration in the filtrate samples were below the assay limit of detection and therefore were excluded from statistical analysis. Total protein concentrations were significantly (p <0.05) lower in acid stimulated saliva compared with resting saliva. There was no significant difference between mechanically stimulated and unstimulated drool saliva concentrations (Figure 1).

**Table 1:** Summary of the concentration levels (median and IQR) for the three analytes and total protein measured in saliva samples collected under resting and stimulated conditions

**Analyte**	**Resting (Unstimulated)**	**Mechanically stimulated**	**Acid stimulated**
**CRP (pg/mL)**	105 (35–217)	97 (32–213)	66 (38–171)
**Myoglobin (pg/mL)**	181 (132–320)	134 (102–202)	147 (111–195)
**IgE (pg/mL)**	142 (56–368)	152 (42–246)	139 (46–221)
**Total Protein (μg/mL)**	1286 (954–2709)	1206 (870–2053)	1026 (821–1680)

**Table 2:** Summary of concentration levels (median and IQR) for the three analytes and total proteins measured in saliva either processed or not processed

**Analyte and sample type**		**Unprocessed**	**Centrifuge**	**Concentrated**
**CRP (pg/mL)**	Unstimulated	105 (35–217)	71 (22–192)	109 (31–282)
	Mechanical	97 (32–213)	48 (20–142)	213 (67–479)
	Acid	66 (38–171)	41 (28–101)	156 (45. - 381)
**Myoglobin**	Unstimulated	181 (132–320)	162 (117–266)	527 (233–650)
**(pg/mL)**	Mechanical	134 (102–202)	111 (82–183)	512 (269–618)
	Acid	147 (111–195)	121 (90–184)	403 (217–591)
**IgE (pg/mL)**	Unstimulated	142 (56–368)	133 (47–382)	155 (57–497)
	Mechanical	152 (42–246)	151 (37–254)	355 (98–711)
	Acid	139 (46–221)	123 (43–197)	193 (84–632)
**Total Protein**	Unstimulated	1286 (954–2709)	776 (369–1319)	2996 (2042–4162)
**(μg/mL)**	Mechanical	1206 (871–2053)	716 (416–1167)	5216 (2921–7813)
	Acid	1026 (821–1680)	736 (491–1153)	4536 (2582–6674)

**Figure 2:**
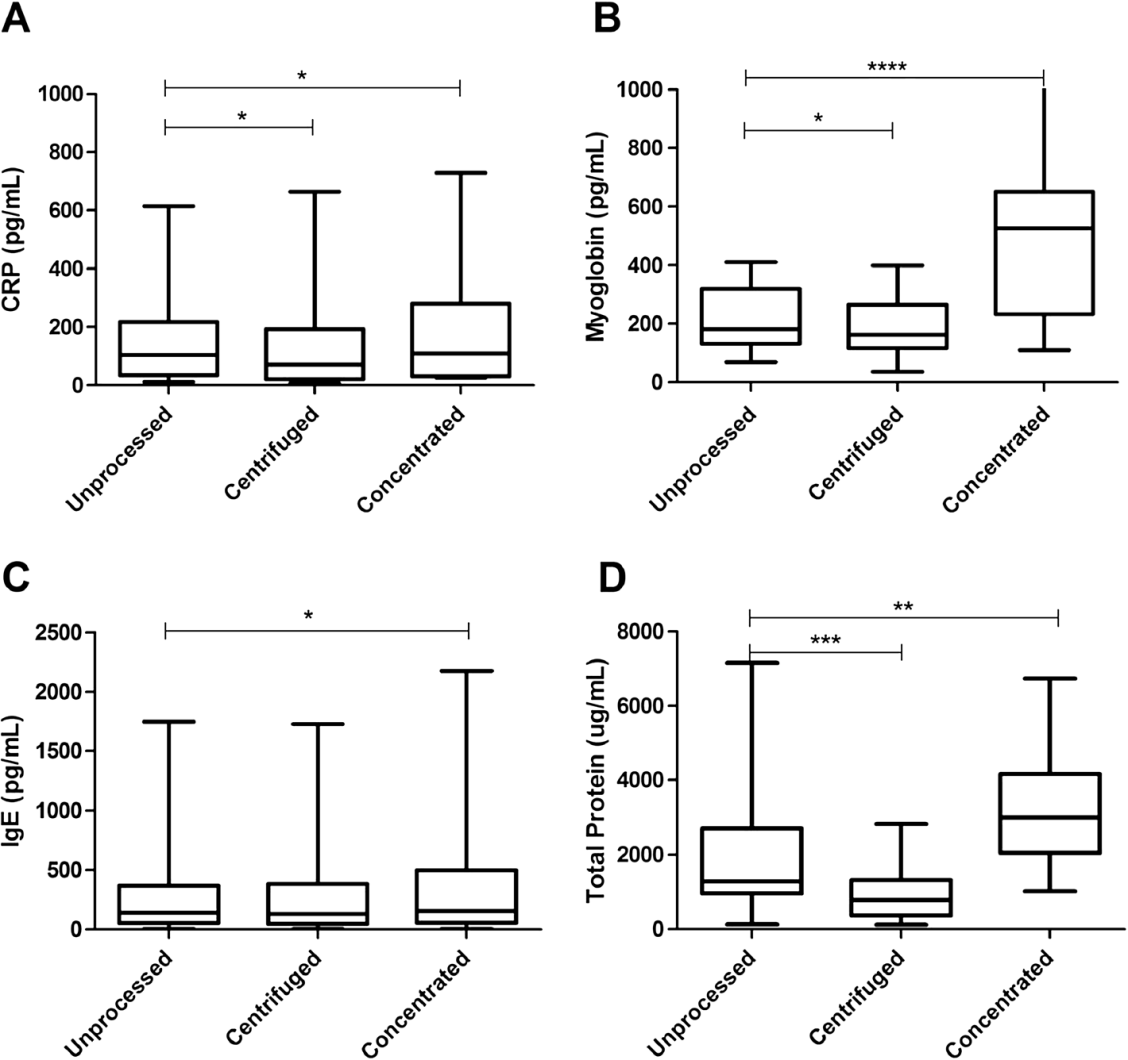
**Concentration of (A) CRP, (B) Myoglobin, (C) IgE and (D) Total Protein in the unprocessed drool, centrifuged drool and molecular weight cut-off concentrate samples. **Minimum and maximum data values are indicated by bottom- and top-most points of box plot. Lower quartile, median and upper quartile values are indicated by first, second and third horizontal lines of the box in the box plot. Significance is shown as *p <0.05, **p <0.01, ***p <0.001, ****p <0.0001.

Centrifugation significantly (p <0.001) reduced the measured total protein concentration in unstimulated drool samples. It is most likely that there is some protein loss during the centrifugation, removing both cellular material and bacterial proteins. Total protein concentration was significantly (p <0.01) higher when saliva samples were concentrated using Centrifugal Filter Units, as opposed to other methods of processing (Figure 2).

### Salivary CRP, myoglobin and IgE in healthy volunteers

The range of CRP, myoglobin and IgE concentrations in the participants is presented in Tables 1 and 2. The concentration of CRP, IgE and myoglobin in the filtrate was below or near the lower level of detection for all three immunoassays and thus excluded from further statistical analysis.

Acid stimulated saliva generally gave lower CRP, myoglobin and IgE levels when compared with resting and mechanically stimulated saliva samples (Figure 1). Resting and mechanically stimulated unprocessed saliva samples gave similar median CRP and IgE levels (Figure 1A &1C), whilst the median concentration of myoglobin measured was significantly (p <0.01) lower in mechanically stimulated, unprocessed saliva when compared with the resting saliva (Figure 1B). Samples concentrated using the Centrifugal Filter Units produced higher levels of CRP, IgE and myoglobin. When saliva samples were centrifuged, a significant reduction (p <0.05) in CRP and myoglobin levels were observed when compared with the unprocessed saliva samples (Figure 2A &2B). However, centrifugation did not affect the concentration of IgE (Figure 2C).

## Discussion

This study demonstrated the effectiveness of saliva as a diagnostic biological fluid depended on the standardisation of the pre-analytical phase, collection and processing methods to deliver the most accurate and meaningful results. Under the prescribed circumstances, we also provided an indication on the likely reference intervals for CRP, IgE and myoglobin in a healthy group of participants. In the present study, we determined the concentrations of total protein, CRP, myoglobin and IgE in human saliva using a homogenous bead-based assay before (unstimulated, drool resting) and after physiological stimulations (acid and mechanically stimulated). The concentration of total protein, CRP and myoglobin were significantly reduced when saliva samples were centrifuged compared with the unprocessed saliva samples. This is likely due to the removal of cells, cellular debris and bacterial proteins during centrifugation process. Similarly, work by [Marshall et al. 28] observed a selective loss in the number of proteins identified on a SDS-PAGE gel post-centrifugation as compared with unprocessed saliva. Therefore, standardization of saliva collection and processing techniques is pivotal to minimising the effect on the variations in saliva composition within and between individuals.

The composition of saliva can vary rapidly according to the flow rate, the type of stimulation and the time of day. Saliva flow rate falls to almost zero during sleep, an important consideration for bedtime snacks and drinks [29]. It is also known that there is great variability in salivary flow rates between individuals [1]. The salivary flow rate for a healthy person’s resting saliva is >0.1 mL/min which is concurrent with our findings (0.52 ± 0.22 mL/min). A person with an unstimulated/resting salivary flow rates below 0.1 mL/min, is considered to have a salivary gland hypofunction [30] and in our current study all of the healthy participants had normal salivary gland functions. This study showed that both methods of swirling citric acid in the mouth and chewing on plastic tubing, significantly increased the salivary flow rates, whilst decreasing the total time required for collecting sufficient volume of sample for downstream applications. These results are comparable with previous work from our group [6]. Besides an increase in the salivary flow rate, the study participants reported that the mechanical stimulation was their preferred method of saliva collection. This infers that mechanical stimulation will be an ideal method to obtain saliva from patients with severe saliva production disabilities, such as in the cases of xerostomia (dry mouth syndrome) and head and neck cancer patients who have undergone radiation [30,31].

It was demonstrated that the concentration of CRP in saliva was similar between resting and mechanically stimulated methods, highlighting the potential use of the latter method in a clinical setting. Similarly, the levels of IgE did not significantly vary between resting and mechanically stimulated saliva, further highlighting the potential use of either method in a clinical setting when determining levels for monitoring immune response [32]. The effectiveness of mechanical stimulation as opposed to other methods of collecting saliva and detecting clinically-relevant proteins has been confirmed in previous studies [33-35]. Mechanically stimulated saliva is known to be predominantly derived from the parotid gland and consists mainly of water [27]. When saliva is mechanically stimulated due to the large volume of water present in this type of saliva, would produce lower proteins concentration when compared with a resting saliva sample [27,36]. This dilution caused by mechanical stimulation appears to have an effect on smaller molecules such as Myoglobin which showed a significantly lower (p <0.01) concentration in the mechanically stimulated samples. However, mechanical stimulation did not appear to dilute IgE or CRP concentrations.

The total protein concentration in normal healthy saliva ranges from 0.5 – 2 mg/mL and this is about 3% of total proteins found in plasma [16]. The resting unstimulated, unprocessed saliva in this study yielded median total protein concentration levels of 1.3 mg/mL. The total protein concentration did not differ between mechanical stimulation and resting saliva, implying that the former method is suitable under circumstances when the patients are suffering from salivary gland hypofunction.

As expected, the use of Centrifugal Filter Units to concentrate salivary proteins represents a viable way to concentrate low abundant proteins in saliva and enable their quantification. One interesting observation from this study is the increase of myoglobin in the concentrated samples, despite myoglobin being theoretically smaller (16.7 kDa) than the micro filter (30 kDa). A possible explanation for this observation is that the myoglobin forms protein complexes with other large salivary proteins and thus is retained in the filters [37]. Salivary myoglobin levels have been used to confirm individuals with Type 2 diabetes and various autoimmune diseases. These groups have elevated myoglobin levels [32].

Saliva sample processing by centrifugation alone has a negative impact on the measured analytes. Centrifugating saliva samples significantly affected the measured levels of CRP and myoglobin, albeit with no significant difference in the measured levels of IgE. It appears that the centrifugation force applied tends to pull the larger proteins down. Gould *et al.* reported that the measured IgE concentrations can be higher in normal healthy controls without signs of allergy [20], and it is possible that some of the participants may have had allergies that they were not aware of or did not disclose in the questionnaire. The natural progression once the collection and processing techniques are established is to look at variability in different age groups, genders, health conditions etc. and investigate the relationship between serum and salivary levels of these proteins for clinical utility.

## Conclusion

In summary, our current study demonstrated that alternative saliva stimulation methods (*i.e.* mechanical stimulation) would be an ideal way to collect saliva in a clinical, challenging environment. Mechanical stimulation showed no effect on the measured CRP and IgE levels thus highlighting the relevance of this method of saliva sample collection. Saliva samples when centrifuged showed significantly lower concentrations of total proteins measured when compared with the unprocessed saliva. Our findings from this research pave the way towards making saliva diagnostics a reality. The standardisation of sample collection and processing method is a step in the right direction in enabling saliva to be studied as a sample of choice for diagnostic purposes where final goal would be to advance the translation of salivary research from a laboratory setting to the bed side of a patient.

## Competing interests

The authors declare that they have no competing interests.

## Author contributions

RM: Experimental execution; JC: Experimental execution; JCW: critical reviewing of the paper; GD: critical reviewing of the paper; CP: design of the study and critical reviewing of the data, analysis; All authors read and approved the final manuscript.
